# Local and Global Breathing Motions Prime the Access to Buried Binding Site in an Antibiotic-Sequestering Protein

**DOI:** 10.1021/acsbiomedchemau.5c00081

**Published:** 2025-08-01

**Authors:** Lawanya Natarajan, Dmitry Loginov, Alan Kadek, Petr Man, Athi N. Naganathan

**Affiliations:** † Department of Biotechnology, Bhupat & Jyoti Mehta School of Biosciences, 37268 Indian Institute of Technology Madras, Chennai 600036, India; ‡ Institute of Microbiology—BioCeV, Academy of Sciences of the Czech Republic, Vestec 252 50, Czech Republic

**Keywords:** protein dynamics, antibiotic resistance, antibiotic sequestration, domain duplication, tryptophan scanning mutagenesis

## Abstract

Proteins frequently undergo large-scale conformational excursions in their native ensemble. Such structural transitions are particularly critical for enabling access to binding sites when they are buried in the protein interior. Here, we map the conformational landscape of AlbAS, a natural isoform of the transcription factor AlbA from the gut microbe *Klebsiella oxytoca*, which sequesters the antibiotic albicidin in a solvent-inaccessible binding tunnel. Combining equilibrium, time-resolved experiments, structural mass spectrometry and calorimetry with statistical modeling, we show that AlbAS displays large differences in local and global stability and dynamics, with ∼600-fold difference in unfolding rates across different parts of the structure. Several residues lining the ligand-binding pocket and the inter-sub-domain residues rapidly exchange protons with the solvent in hydrogen–deuterium exchange mass spectrometry experiments, indicative of anisotropic distributions of local stabilities, with the N-terminal subdomain being less stable. The AlbAS conformational landscape is thus quite rugged, encompassing numerous partially structured states in equilibrium, including partial unlocking of the N-terminal subdomain at a time-constant of 6 ms that exposes the binding sites to aid in albicidin binding.

## Graphical abstract

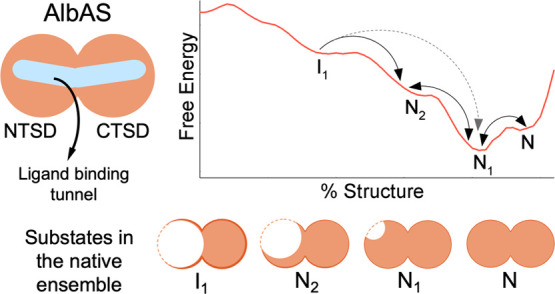

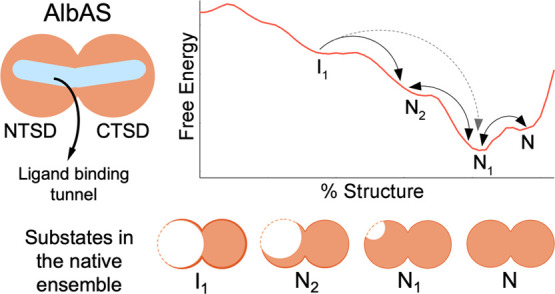

## Introduction

One of the fascinating functions associated with numerous biopharmaceutically important proteins is their role in sequestering antibiotics. Sequestration which involves strong antibiotic binding followed by inactivation is one of the many mechanisms associated with multidrug resistance, which effectively neutralizes the effect of antibiotics.^1,2^ In this regard, *Klebsiella oxytoca*, a common gut microbe, poses serious threats as it evolves into a multidrug resistant (MDR) organism having already acquired resistance against carbapenem, amikacin, colistin, and ceftriaxone. It has been associated with human urinary tract infections, neonatal lung infections, wound infections, sepsis, etc.^3^ Albicidin is a novel drug against gram-negative bacteria with a low minimum inhibitory concentration of ∼40–50 nM, making it a promising alternative to current antibiotics.^4,5^ Albicidin acts by inhibiting the DNA gyrase in prokaryotes, preventing DNA supercoiling and religation, thereby affecting their replication.^6–10^ However, *K. oxytoca* has acquired the gene *albA* that codes for the protein AlbA (Figure 1A), which neutralizes the effect of albicidin by sequestering it and thus reducing the effective intracellular drug concentration.^10^

**Figure 1:**
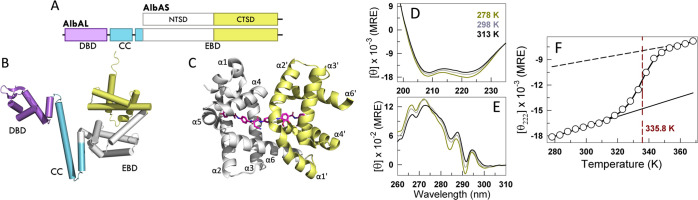
Albicidin binding protein from *Klebsiella oxytoca*. (A) Schematic presentation of the domain organization in two naturally synthesized AlbA isoforms. The long isoform, AlbAL has three domains, while the short isoform AlbAS contains only the effector binding domain, with N- and C-terminal subdomains (NTSD and CTSD, respectively). (B) Cartoon of the structure predicted by AlphaFold2-ColabFold server.^17,18^ DBD, CC and EBD refer to the DNA binding domain, coiled-coil domain and effector binding domain, respectively. (C) Cartoon structure of AlbAS (PDB: 6ET8) with sequestered albicidin. The subdomains (NTSD colored white and CTSD colored pale yellow) each have six helices as labeled. (D,E) Far-UV (D) and near-UV (E) CD spectra at select temperatures. (F) Mean residue ellipticity in units of deg. cm^2^ dmol^–1^ at 222 nm from a far-UV CD thermal melt as a function of temperature (circles) fit to a two-state model (curve through data points). Folded (black solid) and unfolded (black dashed) baselines are also shown. The vertical red dashed line indicates the melting temperature.

AlbA falls under the MerR (Mercuric Resistance) family of transcription regulators,^11–15^ which are characterized by a DNA binding domain, a coiled-coil region, followed by an effector binding domain that binds to toxic ions or drug molecules (Figure 1A). These transcription regulators are primarily dimeric in nature; the binding to effector molecules strengthens their affinity for the promoter region of their own gene, thus enhancing their expression. The translation of the *albA* gene results in the synthesis of two isoforms due to an in-frame translation start site.^10^ The long isoform, AlbAL (40 kDa, 348 amino acid residues) is composed of an N-terminal DNA binding domain, a coiled-coil region that allows dimerization, followed by a C-terminal effector binding domain. On the other hand, the short isoform, AlbAS (25.8 kDa, 221 amino acid residues) contains only the effector binding domain (Figure 1A). AlbA sequesters albicidin through its C-terminal effector binding domain (Figure 1B).

While the complete structure of the long isoform AlbAL has not been experimentally resolved yet, the crystal structure of AlbAS is available (PDB: 6ET8).^10,16^ It is composed of 12 α-helices (Figure 1C), α1–α6 forming the N-terminal subdomain (NTSD) and α1′–α6′ forming the C-terminal subdomain (CTSD), with α6 forming the link between the structural repeats. AlbA binds to albicidin along the entire length of its effector binding domain, through a solvent-occluded substrate binding tunnel (Figure 1C), and with a single albicidin molecule effectively bridging the NTSD and CTSD subdomains. The inaccessible nature of the sequestration site raises questions on the mechanisms through which AlbAS recruits and binds albicidin. In this work, we study the native ensemble properties and dynamics of the apo-form of AlbAS through a combination of experiments and simulations. We show that the AlbAS native ensemble is heterogeneous with multiple substates in which the N-terminal subdomain is partially unfolded. The millisecond time scale access to these conformations potentially enables the binding to albicidin in a manner akin to the conformational selection mechanism.

## Materials and Methods

### Purification

The gene sequence coding for AlbAS from *K. oxytoca* (Uniprot id: Q8KRS7)—MYDRWFSQQELQVLPFAEQDEQRNQTWLELVGEAQQLMGERCPADEPRAIALATRWMEQLEQDTAGRPEFLTRLNEMHAAEPQMREQTGVTPEMIDFITRAFAESKLAIWARYLNAEELAFTRQHYFDRLMEWPALVADLHRACREKRDPASPEGQQLAQRWLALFQSYAGKDAQTQQKFRYAMEQEPHLMKGTWMTSEVLSWLQQAIGVMMRQAQGPAAEA—cloned in pTXB1 vector, between the restriction sites NdeI and SpeI, was purchased from GenScript USA Inc., with a C-terminal intein tag, that enabled IMPACT (Intein-Mediated Purification with an Affinity Chitin-binding Tag) effectuated purification of recombinant proteins. An additional Ala residue was added at the end of the naturally occurring sequence (with Glu as the last residue) increasing the cleavage efficiency and yield of pure AlbAS. Single-tryptophan residue mutants were generated using PCR-mediated site-directed mutagenesis on AlbAS, which had all eight tryptophan residues replaced with phenylalanine, with the aid of primers designed through NEBaseChanger tool and Q5 Hot Start High-Fidelity DNA Polymerase (New England Biologicals).

Recombinant *E. coli* cells expressing wildtype or mutated AlbAS were cultured in Luria–Bertani (LB) broth until an OD_600_ of 0.8–1.0 and induced with 1 mM IPTG. The cells were harvested and resuspended in 200 mM sodium phosphate buffer at pH 8 for cell lysis by sonication. The cell lysate was centrifuged at 10,300 rpm for 60 min at 4 °C. The cleared lysate was loaded into manually packed affinity chromatography columns pre-equilibrated with lysis buffer. The flow rate was maintained between 0.5 and 0.7 mL/min. The column was washed with the same buffer and then filled with the cleavage buffer containing 100 mM β-mercaptoethanol to cleave fusion protein from the intein tag. The column was sealed and incubated at room temperature for 14–16 h before eluting with cleavage buffer at the rate of 0.8–1 mL/min and further applied onto a fresh chitin pre-equilibrated column. The flow was maintained at a rate not more than 0.5 mL/min and the column flow-through was collected to remove free intein and fusion protein that might have eluted along with cleaved AlbAS. The collected flow-through fractions were injected into a 26/10 HiPrep desalting column (Cytiva) pre-equilibrated with 150 mM ammonium acetate at pH 8. The eluted pure fractions were pooled for lyophilization.

### Sample Preparation for Experiments

For all experiments, lyophilized AlbAS was dissolved in a buffer composed of 20 mM sodium phosphate and 107 mM sodium chloride, at pH 7 with an ionic strength of 150 mM. Once dissolved the solution was filtered with a 0.22 μm syringe filter. Concentrations were measured with Jasco V-730 UV–vis. spectrophotometer, employing an extinction coefficient (ε) of 51,450 M^–1^ cm^–1^ for wt and 12,950 M^–1^ cm^–1^ for the W mutants at 280 nm.

### Circular Dichroism

Far- and near-UV CD spectra of the proteins were acquired in a Jasco J-815 spectrophotometer and Applied Photophysics Chirascan-Plus qCD instrument, respectively. The spectra were acquired for every 5 K with an equilibration time of at least 2 min at every temperature, from 278 to 368 K, between 190 and 250 nm for far-UV CD, and between 260 and 320 nm for near-UV CD. Two-state fits to the far-UV CD unfolding curves were performed with six floating parameters, two determining the melting temperature (*T*_m_) and the enthalpy of unfolding at the *T*_m_ (Δ*H*_m_), and two each for the linear folded and unfolded baselines (slope and intercept).

### Steady-State Fluorescence Spectroscopy

Fluorescence measurements of the proteins (4.7 μM of wt and ∼10 μM of mutants) at varying temperatures were obtained in an Applied Photophysics Chirascan-Plus qCD instrument. N-acetyl-tryptophanamide (NATA) at a concentration of ∼9–10 μM (dissolved in HPLC grade water) was used as a reference to calculate the quantum yield. Emission spectra were collected between 300 and 550 nm when the proteins were excited at 295 nm, and between 280 and 550 nm when excited at 274 nm. For urea-mediated unfolding experiments, fluorescence emission spectra were recorded for AlbAS dissolved in experimental buffer containing different concentrations of urea from 0 to 7 M in 0.3 M intervals and at three temperatures 286, 298 and 310 K.

### Fluorescence Lifetime Measurements

Fluorescence lifetimes of tryptophan residues were measured in ChronosBH time-resolved spectrometer, with Ludox solution to measure the instrument response function. AlbAS (wt at 1.4 μM, mutants at ∼10–12 μM) was excited at 300 nm and the time traces were collected at 298 and 313 K. The traces from all proteins were fitted to bi-exponential functions at each temperature point.

### Stopped-Flow Kinetics

Stopped-flow measurements were acquired with a Chirascan single-mixing SF.3 accessory (Applied Photophysics) attached to a photomultiplier tube. The intrinsic fluorescence upon a rapid mixing of protein sample and denaturant was detected by exciting the sample with a 280 nm LED and collecting emitted fluorescence with a 295 nm cutoff filter. Unfolding traces of wt AlbAS was recorded at final urea concentrations between 2.7 and 8 M (for mutants the final concentration was 6 M urea) and a protein concentration of ∼10 μM. Three scans of 1000 data points were recorded at 298 K for each concentration of urea and averaged, before fitting to mono- or multi-exponential functions to extract amplitudes and observed rates.

### Hydrogen–Deuterium Exchange Mass Spectrometry

H/D exchange reactions were set up using a PAL DHR autosampler (CTC Analytics AG) controlled via the Chronos software (AxelSemrau). A 10 μM protein solution in the original sodium phosphate buffer (ionic strength of 150 mM at pH 7.0) was diluted 10-fold with a corresponding D_2_O-based assay buffer at pD 7.0. HDX was monitored for 20 s, 1 min, 5 min, 20 min and 2 h, with each time point done in triplicate. Quenching of the exchange reaction was achieved with chilled 1 M glycine-HCl at pH 2.3, added at a 1:1 (v/v) ratio. Samples were immediately injected into the LC system placed in a temperature-controlled box (0 °C) coupled to an Agilent Infinity II 1260/1290 UPLC (Agilent Technologies) which was directly interfaced with the ESI source of timsTOF Pro equipped with PASEF (Bruker Daltonics). The LC setup, consisting of a pepsin/nepenthesin-2 column (AffiPro, CZ), a trap column (SecurityGuard ULTRA Cartridge UHPLC Fully Porous Polar C18, 2.1 mm ID; Phenomenex) and an analytical column (Luna Omega Polar C18, 1.6 μm, 100 Å, 1.0 × 100 mm; Phenomenex), was cooled to 0 °C to minimize back-exchange. Proteins were digested, and peptides desalted by 0.4% formic acid (FA) in water delivered by a 1260 Infinity II Quaternary pump at 200 μL/min^–1^. To elute and separate the desalted peptides, a gradient of water–acetonitrile (ACN) was used (10–45%; solvent A: 0.1% FA in water, solvent B: 0.1% FA, 2% water in ACN) followed by a step to 99% B, with the solvents being pumped by the 1290 Infinity II LC system at 40 μL/min^–1^. The mass spectrometer was operated in MS mode with a 1 Hz data acquisition rate without using the ion mobility separation. Fully deuterated controls were prepared to perform correction for back-exchange (deuterium loss during the analysis) as described previously.^19^ Acquired LC–MS data were peak picked and exported in DataAnalysis (v. 5.3, Bruker Daltonics) and further processed using DeutEx software (Bruker Daltonics).^20^ Data visualization was performed using MSTools^21^ (https://peterslab.org/MSTools/index.php) and PyMOL version 2.0.6 (Schrödinger, LLC). For peptide identification, the same LC–MS system as described above was used, but the mass spectrometer was operated in data-dependent MS/MS mode with PASEF active and tims enabled. The LC–MS/MS data were searched using MASCOT (v. 2.7, Matrix Science) against a custom-built database combining a common contaminant database cRAP.fasta (https://www.thegpm.org/crap/) with the sequences of AlbAS and the utilized proteases. Search parameters included: precursor tolerance 10 ppm, fragment ion tolerance 0.05 Da, decoy search with FDR <1%, IonScore >20, and peptide length >5. The mass spectrometry proteomics data have been deposited to the ProteomeXchange Consortium via the PRIDE^22^ partner repository with the data set identifier PXD061296. The average deuteration of each amino acid or stretch of amino acids was calculated as described in a previous work.^100^ The structural boundaries of helices in AlbAS, which we used as the primary structural feature for all our analyses, do not, of course, exactly match with the proteolytic peptide segments identified in the HDX experiments. The segments for calculating the averaged percentage deuteration values ⟨%*D*⟩ of each helix were thus chosen to maximize this overlap. Similarly, the number of segments that result in the averaged values also vary somewhat between individual helices, as described in detail in Table S1 in the Supporting Information. The apparent equilibrium constant ln­(*K*_app_) is calculated from

$$\ln\left(K_{app}\right)=\ln\left(\frac{p_{D}}{p_{H}}\right)=\ln\left(\frac{p_{D}}{1-p_{D}}\right)$$

where *p*_D_ is the deuterated fraction of a peptide segment and *p*_H_ is the non-deuterated fraction at a time point of 20 s, i.e. the earliest time point accessed. Note that the trend in ln­(*K*_app_) is equivalent to that of Δ%*D*.

### Differential Scanning Calorimetry

Highly concentrated samples of wt AlbAS were filtered and desalted with 26/10 HiPrep column from Cytiva, to eliminate trace residual salts from the protein after lyophilization, and to enable accurate heat capacity measurements. Samples were degassed before loading into the calorimetric cells. DSC profiles were acquired in a Microcal VP-DSC instrument at a scan rate of 1.5 K/min at different concentrations (96.2, 104, and 124.1 μM), and with buffer sans protein in reference cell. Multiple buffer–buffer baselines were acquired before and after every protein scan to check for baseline drifts. The apparent heat capacities were then employed to estimate the absolute heat capacity via the method proposed by Sanchez-Ruiz and co-workers.^23^

### Block Wako–Saitô–Muñoz–Eaton (bWSME) Model

The model is described in detail in a recent work.^24^ In short, like the original WSME model^25,26^ that considers every residue as a folding unit (assigned a binary variable 1 for folded and 0 for unfolded), we have developed a “block” version wherein stretches of consecutive residues are assumed to fold as a single block to reduce the number of constituent microstates without compromising model predictions.^27^ In this work, we employ a block-size of 3 and account for only those microstates that fall within the following approximations—single sequence approximation (SSA; a single stretch of folded blocks), double sequence approximation (DSA; two folded stretches separated by unfolded blocks), DSA with loop (DSAw/L; DSA but allowing for interactions across folded stretches). The free energies and hence the statistical weights (*w*) of the microstates are derived from a native-centric treatment starting from the PDB structure with contributions from van der Waals interactions (5 Å heavy-atom cutoff), all-to-all Debye–Hückel electrostatics, solvation and secondary-structure-dependent conformational entropy (Δ*S*_conf_) with an excess entropic penalty ΔΔ*S* of −6.1 J mol^–1^ K^–1^ for glycine residues and residues in coil regions. The total partition function (*Z*_T_) is calculated by summing up the statistical weights of microstates, from which the heat capacity curves, free-energy profiles and coupling free-energy matrices are derived.^28^ The residue-level local stability is calculated from Δ*G*_s_ = −*RT*ln­(*p*/(1 – *p*)) where *p* is the probability of the residue to be folded. Here, we perform a global trial-and-error fit considering both the DSC profile (a measure of global stability) and the HDX-MS data (measure of local stability, i.e. ln­(*K*_app_); see the section above) to calibrate the model parameters systematically. The final parameters from this exercise are the van der Waals interaction energy of −36.0 J mol^–1^ per native contact and an entropic penalty per residue of −7.0 J mol^–1^ K^–1^. In addition, the experimental data required that the contact-maps of the residue stretches 1–26, 62–84, 168–197 are weakened to 0.7, 0.66, and 0.7 of their original values, while those of the stretches 27–55 and 201–209 are strengthened by 1.2 and 2.3, respectively. The codes used in this work are available at: https://github.com/AthiNaganathan/WSMEmodel.

## Results

### Anisotropic Distribution of Local Packing and Stabilities

Far-UV CD spectra of AlbAS (Figure 1D) at selected temperatures exhibit the characteristic negative peaks at 208 and 222 nm, consistent with the helical structure reported in the experimental crystal structure.^10^ Fine spectral bands are also evident in near-UV CD spectra, indicating that the protein is not a molten-globule (Figure 1E). In fact, AlbAS harbors multiple aromatic residues - nine phenylalanine, five tyrosine, and eight tryptophan residues – distributed through the structure. The melting curve obtained by measuring the CD signal at 222 nm as a function of temperature (Figure 1F) appears two-state-like, with a melting temperature of 335.8 ± 0.3 K from a two-state fit, albeit with steep pre-transition baselines indicative of structural changes in the physiological range of temperatures (<315 K).

AlbAS has eight tryptophan residues, four in each subdomain. Out of the 12 helices that constitute the 222 residues long structure of AlbAS—α1–α6 (NTSD) and α1′–α6′ (CTSD)—every helix has a tryptophan residue (Figure 2A), except for helices α4, α5, α4′ and α5′. While tryptophan residues can provide information on the degree of local polarity (either via exposure to solvent or through interaction with other polar moieties within the protein^29^), the presence of multiple tryptophan residues masks their individual contributions in fluorescence-based experiments. To probe the local structural environment more precisely across the protein, mutants with a single tryptophan residue were generated. For this, the tryptophan residues are replaced with phenylalanine (a conservative replacement) in all locations except one which acts as the sole fluorescence probe. For instance, the mutant named W5 has tryptophan residues replaced with phenylalanine residues in all sites except at the fifth position. Thus, eight such AlbAS mutants with single tryptophan residues were generated.

**Figure 2:**
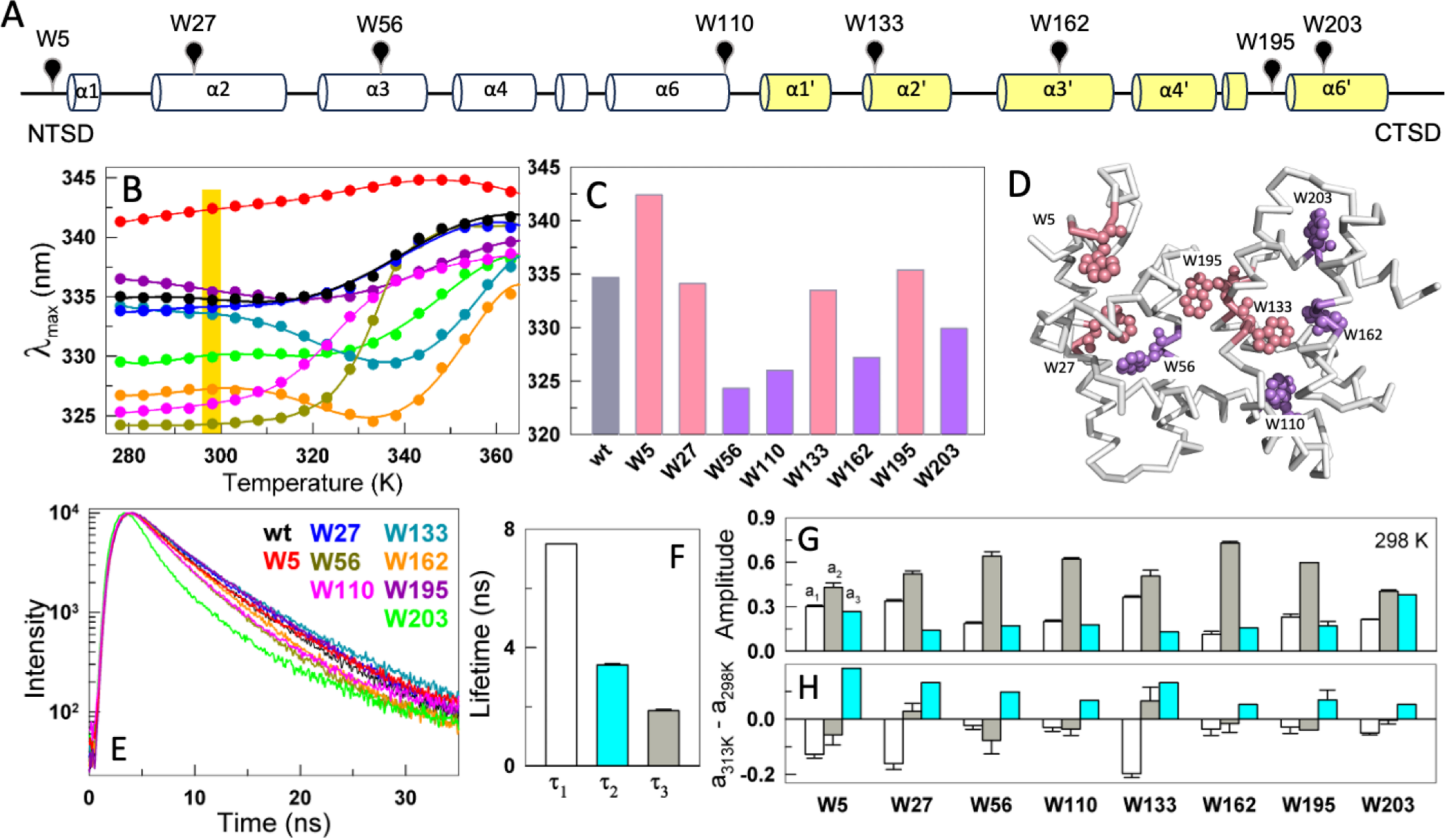
Heterogeneity in local stability and dynamics. (A) Location of tryptophan residues on the structure of AlbAS in a linear schematic with cylinders representing helices. NTSD and CTSD are colored white (α1–α6) and yellow (α1′–α6′), respectively. (B) Fluorescence emission maxima of wildtype and single-tryptophan mutants shown as circles, while curves are shown to guide the eye (color-coded as in panel E). The λ_max_ values highlighted in yellow at 298 K span about 18 nm, from 324 to 342 nm. (C) λ_max_ at 298 K from panel B, with the wt shown in gray. Relatively more solvent-exposed tryptophan residues (relatively higher λ_max_) are colored pink while those buried (lower λ_max_) are colored purple. (D) Tryptophan residues highlighted on AlbAS, colored according to panel (C). Five of the eight tryptophan residues (W27, W56, W133, W162, and W195) point toward or line the ligand-binding tunnel. (E) Fluorescence intensity decays of the wt and mutants at 298 K. (F) The decay curves in panel (E) are best fit to bi-exponential functions to extract the lifetimes and corresponding amplitudes, with the mean of the two lifetimes extracted from all the mutants shown. (G) Amplitudes corresponding to each of the lifetime components are disparate across mutants, pointing toward a non-uniform structural packing and large variation in local nanosecond dynamics. (H) Amplitude differences between 313 and 298 K showcase the enhancement of local nanosecond dynamics, with the amplitude of the shorter lifetime (indicative of a more fluid local environment) increasing at the expense of the longer lifetime amplitude.

The thermal unfolding curves of the tryptophan mutants observed via far-UV CD are shown in Figure S1A. The melting curves have similar pretransition slopes, with the melting temperature (*T*_m_) and the enthalpy of unfolding at the midpoint (Δ*H*_m_) estimated from two-state fits displaying a spread of 12 K and 63 kJ mol^–1^, respectively (Figure S1). Only W110 (i.e., W at position 110 and phenylalanine in other tryptophan positions) and W195 destabilized the protein by >7 K, with the rest of the mutations having a marginal effect. Interestingly, the CD signal of W5 is lower (more negative) than other mutants or wildtype; this likely results from the variable absorption of aromatic residues at ∼222 nm which is also dependent on the degree of helicity at that site, thus distorting the estimation of actual helical content in the protein.^30^

In stark contrast to far-UV CD melting curves, the steady-state fluorescence thermal melts of the mutants result in more varied profiles (Figure 2A,B). A few trends are apparent from these measurements. First, the residues W56, W110, W162 and W203 are observed to be in an apolar environment at 298 K (λ_max_, fluorescence emission maximum, ∼324–330 nm) while W5, W27, W133, and W195 are in a more polar environment with a λ_max_ ∼333–342 nm (Figure 2B,C). The differences, which are apparent even at the lowest temperatures, report that the tertiary structure of AlbAS is not uniformly packed with varied solvent exposure of the tryptophan residues. Second, W5, W195 and W27 reveal only minor changes in λ_max_ (<5 nm) across the entire range of temperatures, hinting that these residue sites are partially unfolded even at the lowest temperatures. Third, residues W133, W162 and W203 reveal a *T*_m_ > 345 K despite only minimal changes of stability relative to the wt from far-UV CD measurements (i.e., *T*_m_ within 3 K of the wt; Figure S1). This specific observation, independent of the local polarity, signifies that the CTSD (in which the latter three tryptophan residues are located) is more stable than the NTSD. Fourth, the two probes in the CTSD—W133 and W162—display a decreasing λ_max_ with temperature until ∼330 K following which they unfold. This highlights the presence of a potential intermediate state in which the CTSD exhibits an altered packing density upon melting of the NTSD. Finally, the relatively higher polarity around W195 (dark pink in Figure 2B), which is located right at the interface between the two subdomains, and the minimal changes in λ_max_ with no apparent unfolding curve are evidence that the intersubdomain interface is only weakly packed.

### Heterogeneity in Local Dynamics

Steady-state fluorescence measurements hint at substantial variation in the three-dimensional packing of the structure at different locations of the protein. This is further probed by time-resolved fluorescence measurements, which also report on the environment around tryptophan residues, but from the perspective of lifetimes and their amplitudes. Lifetimes report on the torsional rigidity (either from the indole alone or in conjunction with the protein backbone) with longer lifetimes indicative of a more rigid environment.^31^ The amplitudes, on the other hand, quantify the fractional contribution of different modes.

The fluorescence lifetime traces at 298 K (Figure 2E) are best fit to a bi-exponential function (Figure S2A,B), leading to two lifetimes and amplitudes. Figure 2F plots the extracted lifetimes from the single-tryptophan mutants, with a longer lifetime (τ_1_) of 5.6 ± 0.2 ns pertaining to a more folded-like environment, and a shorter lifetime (τ_2_) of 2.1 ± 0.1 ns suggestive of a more unfolded-like environment. The amplitude-weighted lifetime ranges between 2.9–4.8 ns, with a mean of 4.1 ± 0.03 ns, explaining the similarity in the lifetime traces among mutants (Figure S2C). On the other hand, the amplitudes corresponding to the lifetimes at 298 K exhibit great variation across the mutants (Figure 2G). Interestingly, none of the longer lifetime amplitudes are high, i.e. not >0.8 that is expected of a well-folded environment,^32^ indicating that the interior of AlbAS is quite fluid. Upon increase in temperature, the amplitude of the longer lifetime decreases while that of the shorter lifetime increases, signaling an enhanced torsional mobility of the side-chains or the peptide backbone. If AlbAS behaves as a uniformly rigid molecule, then the extent to which the amplitudes change across the structure is expected to be uniform since the lifetime experiments are performed at low temperatures, well below the respective melting temperatures. However, the observed heterogeneity in amplitudes between different mutants and across the two lifetimes showcases that the local environment determining the extent of torsional mobility around the tryptophan varies at each location, potentially as a precursor to global unfolding. Furthermore, the tryptophan residues in the CTSD (W162, W195, W203) appear to undergo larger amplitude changes compared to those at the NTSD (except for W5).

### Site-Specific Multistate Unfolding

The evolution of structural changes in AlbAS upon rapid mixing of urea is studied through stopped-flow fluorescence kinetics. Here, two solutions in two syringes—one containing AlbAS in buffer and the other containing 6.6 M urea—are mixed within 3 ms (the final urea concentration will be 6 M), and the time evolution of the loss of structure is monitored as a function of time. The kinetic traces obtained for wildtype AlbAS fit best to a bi-exponential function (Figure 3A–D), thereby yielding two observed rates at every urea concentration (Figure 3E). This observation qualitatively rules out a two-state model of unfolding and is proof for the presence of an intermediate. The observed rates are quite slow ranging from 2–30 s^–1^ over the range of urea concentrations studied, with the faster rate displaying a shallow chevron-like behavior and the slower-rate exhibiting a rate independent of [Urea] below 5.5 M (Figure 3E). The amplitude of the slower phase increases with [Urea], and it therefore reports on the population of the unfolded state (Figure 3F). On the other hand, the amplitude of the faster phase increases with [Urea], peaks at 6–7 M urea, and decreases at higher concentrations. This is a signature feature of an intermediate whose population increases upon destabilization, and decreases at high [Urea] due to exchange with the unfolded state.

**Figure 3:**
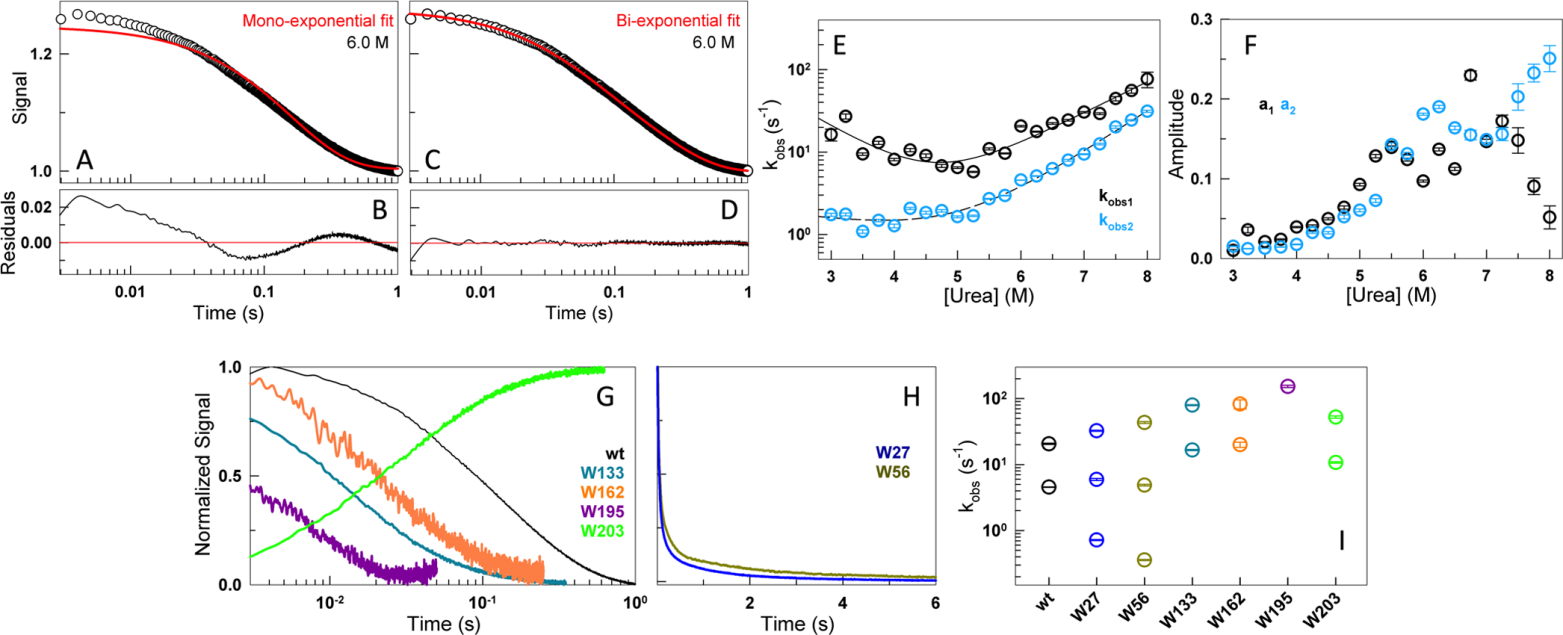
Site-specific multistate unfolding. (A–D) Fluorescence intensity traces from stopped-flow experiments at a final concentration of 6 M urea for the wt, fit to mono-exponential (panel A) and bi-exponential (panel C) functions, with the corresponding residuals (panels B and D, respectively) shown below. (E) The urea dependence of the two observed rates. (F) Amplitudes corresponding to the observed rates in panel E. (G) Normalized unfolding traces of W133, W162, W195 and W203 at 6 M urea, in comparison with the wt. (H) Normalized unfolding traces of W27 and W56 on a linear time scale to highlight the slowest component. (I) Observed rates extracted from fitting the kinetic traces of the mutants and the wt at 6 M urea to a mono-, bi- or tri-exponential functions (refer Figure S3).

The observed fluorescence intensity traces in the wildtype protein are an effective average of the signals from the eight tryptophan residues, and thus do not shed light on the possibility of site-specific differences in unfolding rates. To explore this, we carried out unfolding kinetic measurements at 6 M urea on the tryptophan mutants at 298 K. Remarkably, the mutants exhibit distinct relaxation profiles (Figure 3G,H), with time ranges substantially shorter and longer than those observed for the wildtype protein. Fluorescence intensity traces of two of the mutants, W5 and W110, did not lead to any appreciable change in intensity and hence were not studied further. Among the other six mutants, the unfolding of the W195 variant is best explained by a single observed rate constant (*k*_obs_), W133, W162 and W203 required two rate constants, while W27 and W56 necessitated the use of three *k*_obs_. The *k*_obs_ range from 0.4 s^–1^ (slowest rate of W56) to 152.3 s^–1^ (W195), thus spanning nearly 3 orders of magnitude.

The fastest relaxation is observed in the mutant W195, which also happens to report on the interface between the two subdomains; it is located on the loop connecting helices α5′ and α6′ (in CTSD) but spatially close to the helices α3 and α4 in the NTSD. This illustrates that the two subdomains undergo partial opening events with a time constant of ∼6 ms as sensed by W195. The change in the environment around W195 could, however, arise from either of the domains unfolding or both undergoing coordinated domain-opening events. The very slow relaxation rates observed in W27 and W56 (<1 s^–1^) signal that some regions in NTSD unfold slowly, while the rest of the NTSD undergoes relatively faster relaxation. In summary, the existence of multiple phases with large differences in the associated rate constants paints a picture of an intrinsically rugged unfolding landscape in AlbAS, consistent with the site-dependent tryptophan solvent accessibility from steady-state fluorescence and varying packing extents from time-resolved fluorescence measurements.

### Local Stability Profiles from HDX-MS

To further corroborate our data using an orthogonal technique not reliant on mutagenesis and to better elucidate residue-level differences in local stability of AlbAS, we used hydrogen–deuterium exchange mass spectrometry (HDX-MS).^33,34^ HDX-MS monitors the dynamics and the level of structuring in regions of protein by probing backbone amide hydrogens for their solvent accessibility and involvement in hydrogen bonding. This is achieved by incubating proteins in a buffer prepared with D_2_O, in which backbone amides spontaneously exchange for deuterium resulting in a detectable mass increase. This is monitored across a range of incubation times providing a comprehensive time-resolved view of the extent to which various sections of the protein are amenable to exchange with the solvent and hence report on local stability and dynamics. Even during the shortest incubation time point in our experiments (20 s), many residues in the NTSD exchange fully; these include residues 1–25 (α1 and sections of α2), 60–85 (α4 and α5), and the C-terminal end of CTSD including residues 210–222 (C-terminal residues of α6′) (Figure 4A,B).

The regions spanning α2 and α3 in the NTSD exchange extremely slowly with only minimal deuteration over the 2 h period, which is also mirrored in the structurally similar α2′ and α3′ of the CTSD. The other regions of CTSD exhibit moderate exchange in helices α1′, α4′, and α5′ at 20 s and a steady increase in %*D* in all helices with incubation time. The average deuteration extents for the different helical regions and at various time points (Figure 4C,D and Table S1) report on the structurally labile nature of NTSD, with the CTSD exchanging slowly over the 2 h period. These trends can be vividly observed upon mapping the %*D* onto the structure (Figure 4E,F).

**Figure 4:**
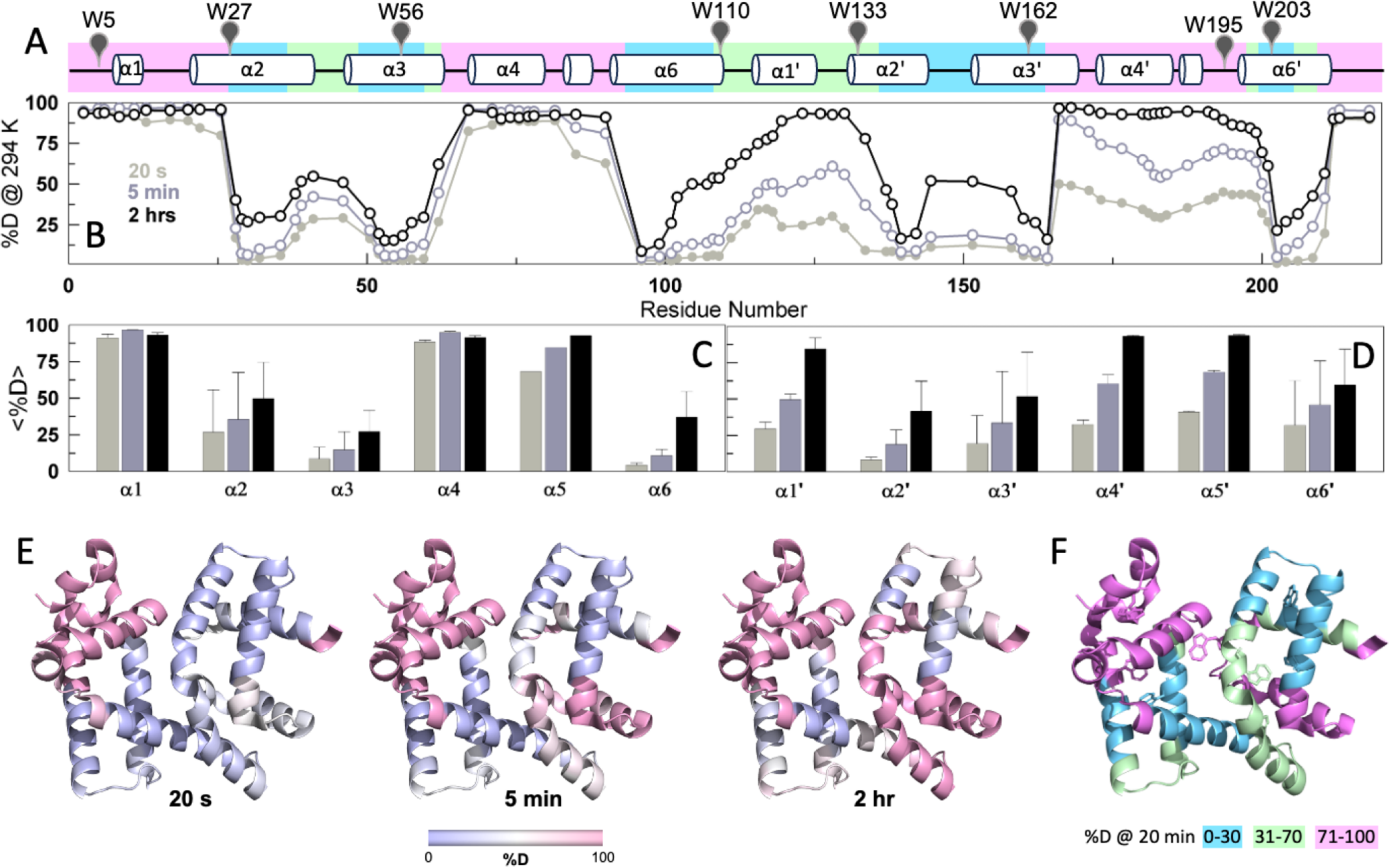
HDX-MS reveals distinct local stability profiles. (A) Schematic of secondary structure organization, highlighted by colors corresponding to the extent of hydrogen exchange, with pink, green, and blue indicating high, moderate, and low exchange at 20 min. (B–D) Percentage deuteration (%*D*) across protein length after 20 s, 5 min, and 2 h of incubation in deuterated solvent (panel B). The NTSD of AlbAS has greater exchange at 20 s than CTSD. Grouped bars showing averaged %*D* across each helix in NTSD (panel C) and CTSD (panel D) at select time points as panel B. Error bars indicate standard deviation in averaging across segments comprising the helices. A steady increase in %*D* with incubation time is seen in helices of CTSD. (E) %*D* values of peptide segments mapped onto AlbAS structure at different time points. The spectrum ranges from blue to pink (color-bar shown below) for increasing %*D* recorded. (F) Cartoon of AlbAS structure showing tryptophan (sticks) colored according to %*D* values at 20 min following colors scheme described below (same as panel A).

Experimental observations made on the wildtype through HDX-MS complement those on tryptophan mutants from equilibrium fluorescence experiments. At 20 min, peptides with W5, W27, W133, and W195 exhibit >50% deuteration (Figure S4A), and this observation matches well with high solvent accessibility of these tryptophan residues in fluorescence experiments (highlighted by light pink in Figure S4B). Peptides harboring W56, W110, W162, and W203 exhibit low deuteration extents (<50%) at 20 min, and this again is in accordance with the low solvent accessibility extracted from fluorescence measurements on the mutants (highlighted by purple in Figure S4B). More than three-fourths of the residues that have been identified to form the hydrophobic core of the ligand-binding tunnel in AlbAS display moderate to high exchange with deuterated solvent (Table S2). Specifically, nearly 60% of the residues in the ligand-binding site of the NTSD are >60% deuterated with median deuteration extents of 80% even at the shortest exchange time (20 s), while the corresponding residues in the CTSD are only <50% deuterated (median deuteration extent ∼26%). These observations point to a dynamic equilibrium between open (exchange-competent) and closed (not exchange-competent) conformations in several residues lining the binding site in the NTSD.

In addition, the IUPred3 ^35^ and pLDDT (from AlphaFold2 ^17^) scores mirror experimental HDX-MS trends for most parts (Figure 4B is reproduced in Figure S4D for direct comparison). Specifically, the predicted disorder scores from IUPred3 in the short-disorder mode (Figure S4E, red curve) show the first five and last ten residues of the protein to be disordered, along with the helices α4 and α5 (scores >0.5) that matches the results from HDX-MS. The helices α6, α1′ and α2′ (residues 100–140) are also predicted to be the most ordered, which in fact exhibit the least deuteration in HDX-MS. Predicted local distance difference test (pLDDT) scores from the AlphaFold2-ColabFold Web server^17,18^ can also be employed as a measure of expected local or global disorder. For AlbAS (Figure S4E, blue curve), the pLDDT scores are low for the first 30 residues (α1) and for residues corresponding to α4 and α5, which aligns well with a high %*D* displayed by these regions even at 20 s. However, pLDDT scores are close to 100 for most of the CTSD, except for a steep decrease beyond 210 residues, indicative of a more ordered CTSD compared to NTSD.

### The Native Conformational Landscape of AlbAS

Given the decoupled unfolding of different structural elements in AlbAS, it is expected that AlbAS should undergo large enthalpic fluctuations (σ_H_^2^) in the native ensemble. To quantify this, we measured the absolute heat capacity profile, *C*_p,abs_, of AlbAS from the concentration dependence of apparent heat capacity (Figure S5A). The resulting heat capacity profile exhibits a sharp transition with a peak heat capacity temperature of 337 K (Figure 5A), but which cannot be well-described by a two-state model due to the crossing of folded and unfolded baselines (Figure S5B). The Freire baseline is an empirical estimate of the enthalpic fluctuations expected of a fully folded globular protein.^36,37^ The fact that the pretransition region of AlbAS has much higher heat capacity value than what is expected of fully folded proteins at even low temperatures (<315 K) is further strong evidence that AlbAS does not have a very compact structure. Using the known thermodynamic relation connecting heat capacity to enthalpic fluctuations,^38–40^ *C*_p_ = σ_H_^2^/*RT* ^2^ and at *T* = 298 *K*, we find that the AlbAS displays ∼22% more enthalpic fluctuations than that expected of a well-folded protein.

**Figure 5:**
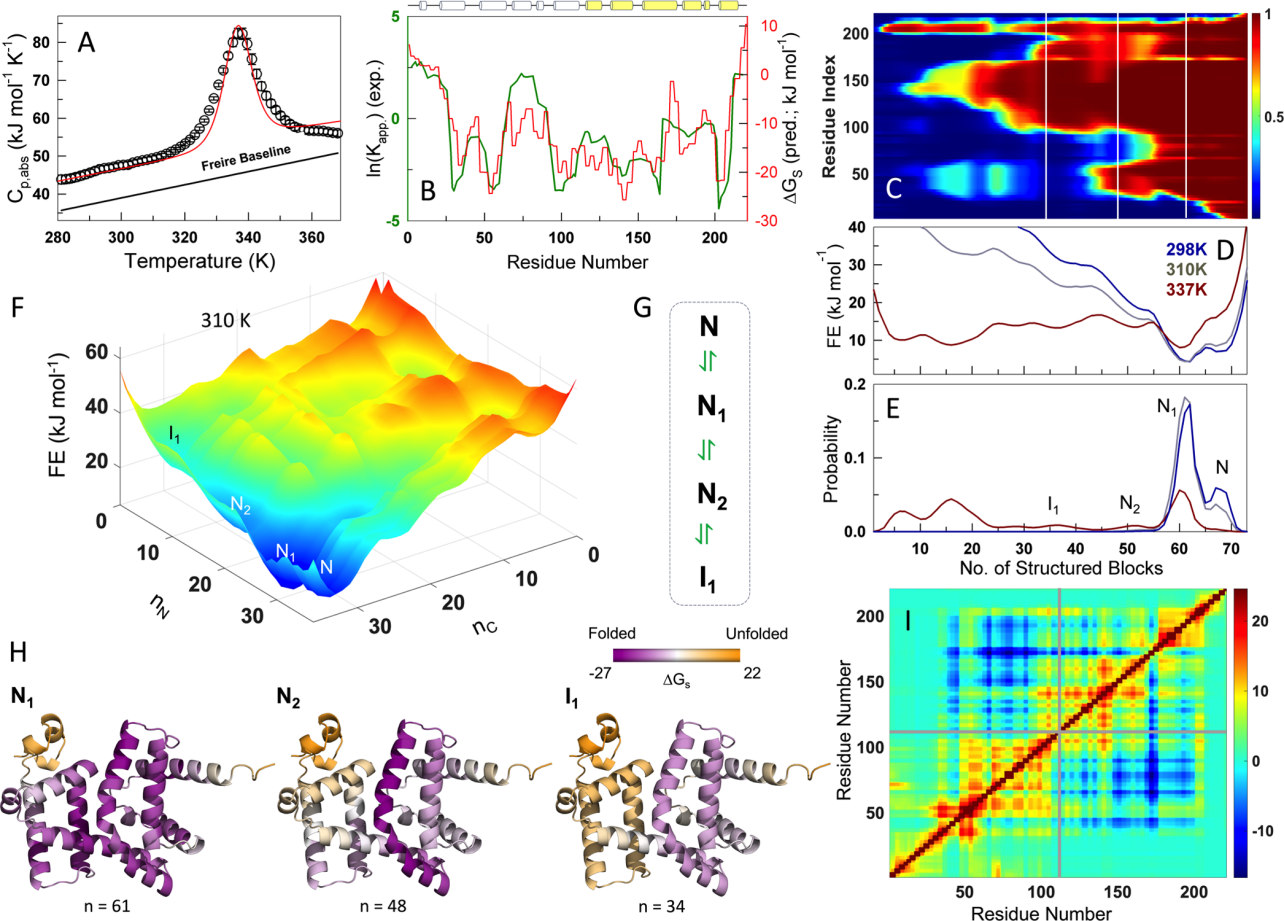
Native conformational landscape of AlbAS. (A) The DSC thermogram of wt AlbAS (black circles) fit to the block WSME model (red curve). The Freire baseline (black line) highlights the expected fluctuations in a fully folded protein. (B) The logarithm of the apparent equilibrium constant, ln­(*K*_app_), calculated from experimental HDX-MS data (green) is compared with the local residue-level stability predicted by the bWSME model (Δ*G*_s_, right axis). The cartoon on top represents the helices in the NTSD (white) and CTSD (yellow). (C) Two-dimensional heatmap of the residue folding probability as a function of the number of structured blocks (the reaction coordinate, RC) on the *x*-axis and residue index on the *y*-axis. White lines represent macrostates, whose structures are shown in panel H. (D, E) One-dimensional free-energy profiles (D) and the corresponding probability density (E) at select temperatures. (F) Free energy surface plot at 310 K as a function of the number of structured blocks in the N- and C- terminal halves. (G) Schematic of chemical multistate equilibrium between the substates *N*, *N*_1_, *N*_2_, and *I*_1_. (H) Structure of AlbAS representing the substates *N*_1_, *N*_2_, and *I*_1_, colored as a function of local residue-level stability (color bar above in units of kJ mol^–1^). (I) Pairwise effective coupling free energy matrix, Δ*G*_c_, at 310 K. A strong local coupling is observed within each subdomain (demarcated by gray lines), while there is negative coupling between NTSD and CTSD. The colorbar is in units of kJ mol^–1^.

We resorted to modeling the native conformational landscape of AlbAS through the statistical-mechanical block Wako–Saitô–Muñoz–Eaton (bWSME) model.^24,27^ This structure-based approach can quantitatively reproduce the trends in experimental data, enabling a nuanced but experimentally consistent view of the folding conformational landscape. In the current treatment, we consider a large ensemble of 2,258,647 microstates, with the free energy of every microstate determined by contributions from van der Waals interactions, electrostatics, simplified solvation free energy and entropic penalty for fixing residues in the native conformation. The model parameters are tuned to simultaneously reproduce both the heat capacity profile (which provides information on the global stability and cooperativity) and the apparent equilibrium constant from HDX-MS (which is a measure of residue-level local stability; see Methods). From the model perspective, the temperature-dependent heat capacity can be directly calculated from a derivative of the total partition function (*Z*_T_). To reproduce the apparent equilibrium constant from HDX-MS, we employ residue-level local stability (Δ*G*_s_) which is a measure of the degree of foldedness of a residue and that can be explicitly calculated from the bWSME model (see Methods).

The model is able to semi-quantitatively reproduce the sharpness of the DSC curve, its inflection point, and the folded/unfolded baselines (red curve in Figure 5A). In addition, the overall trends observable in HDX-MS data (green in Figure 5B) are also very well captured (red in Figure 5B). The variable, the number of structured blocks, is a natural reaction coordinate (RC) of the model. When the folding probability of every residue is projected onto this coordinate, we find that specific structural regions including α1′, α2′, α3′ and α6′ in the CTSD fold first (at RC < 30; Figure 5C). The folding of the CTSD is followed by partial structuring of many regions in the NTSD as one moves along the RC, with the first 20 residues in the NTSD structuring last. Partial partition functions (*Z*_i_) calculated from the sum of statistical weights of all states with a specific number of structured blocks (*i*) enables the calculation of free energy profiles through the relation *G*_i_ = −*RT*ln­(*Z*_i_/*Z*_T_). The resulting profiles are multistate highlighting two highly populated substates within the native ensemble termed *N* and *N*_1_ (Figure 5D,E); the former is the fully folded state while the latter is a partially structured state in which the first 20 residues (α1 and the N-terminal residues of α2; RC value at *n* = 61) are unstructured. It is interesting to note that *N*_1_ is more stable than the fully folded state by ∼3 kJ mol^–1^ (which is of the order of thermal energy), and therefore in dynamic equilibrium with each other at 310 K.

A third state *N*_2_ (*n* = 48) appears as a sparsely populated excited state^41^ on the free energy profile which is less stable than *N*_1_ by ∼10 kJ mol^–1^. *N*_2_ is characterized by unfolding of the first 30 residues in the NTSD and partial structure encompassing residues 70–100, which correspond to α4 and α5. This is exactly the region in contact with W195 in the CTSD, and partial unfolding in this region determines the solvent-exposed fluorescence properties of W195. Moreover, in unfolding kinetics, this is the first partially structured state that will be populated en route to the unfolded state and hence reports on rapid kinetics compared to other tryptophan probes. Finally, an additional state labeled *I*_1_ (*n* = 34) and which is less stable than *N*_1_ by ∼20 kJ mol^–1^ is also observable in which the entire NTSD is unfolded. At 337 K (the melting temperature), both *I*_1_ and *N*_2_ form a part of the transition state ensemble which is quite broad spanning a large collection of substates with different degrees of structuring in the NTSD. These conformational substates are also evident in the free-energy surface constructed by partitioning the RC into the number of structured blocks in the N- and C-terminal halves of the structure (Figure 5F). Thus, a simple chemical reaction diagram and the associated partial structure in the NTSD is sufficient to capture the dynamic behavior of AlbAS (Figure 5G,H).

To investigate the extent of interactions and associated coupling between NTSD and CTSD, we generated the pairwise effective coupling free energy matrix (Δ*G*_c_) at 310 K (Figure 5I). Coupling free energies are calculated from the ensemble of bWSME model microstates by accounting for pairwise probabilities of all residue-pairs; positive and negative Δ*G*_c_ indicate strong and weak coupling and directly report on correlated and anticorrelated dynamics, respectively.^28^ A strong positive coupling is observed within the subdomains as visualized in the first and third quadrants demarcated by the gray line (Figure 5I). On the other hand, most residues across the two subdomains are negatively coupled (blue in the second and fourth quadrants). The only exception being the residue stretch between 50 and 60 which is a part of α3 that lines the interface between the two subdomains. In other words, despite sharing an extensive interface in the folded structure in the presence of albicidin, the two domains exhibit uncorrelated motions in the native ensemble in the *apo* form. This feature is primarily driven by the weakly folded nature of the NTSD that is in dynamic equilibrium with numerous partially structured states, enabling local and global breathing motions across the protein.

## Discussion

In this work, we combine experimental probes of different structural resolution to map the conformational landscape of AlbAS, an independently expressed and folded isoform of AlbAL. By all measures, AlbAS appears to be a folded helical protein domain with well-defined near-UV CD signals (indicative of tertiary packing), sigmoidal unfolding profiles, and large excess heat capacity in DSC that report on strong cooperativity in the unfolding transition. However, deviations from a simple two-state transition and the underlying complexity emerge at every other global measure. Specifically, a two-state model does not account for the AlbAS DSC curve, the extent of enthalpic fluctuations is substantially higher than that of a well-folded protein, there is a large difference in unfolding enthalpies derived from far-UV CD and DSC (Δ*H*_m_ of 180 kJ mol^–1^ and 305 kJ mol^–1^, respectively), and remarkably, the unfolding enthalpies are less than one-half expected from size-scaling arguments^42^ (645 kJ mol^–1^ from 2.92 kJ mol^–1^ per residue for *N* = 221 residues). These large deviations from a simple cooperative system from different thermodynamic measures are carried over to kinetics as well; the unfolding kinetics is multistate-like with at least two observed rate constants, and whose magnitudes are nearly 100-times faster than that expected from the scaling of folding times with protein length^43^ (i.e., the expected relaxation rate is of the order of 0.01 s^–1^).

The local measures of stability accordingly reveal a highly malleable structure, with rapid side-chain motions affecting tryptophan lifetimes across the entire protein irrespective of the location. The fluid nature of AlbAS interior is also consistent with reports on multiple proteins,^44,45^ and highlights how such dynamics when extended to longer times lead to large-scale unfolding events, akin to observations in the protein barstar.^46^ The lower cooperativity of AlbAS (as measured by DSC) therefore has its origins in the enhanced side-chain dynamics that weaken packing, thus lowering the “heat” required for inducing denaturation. The slower time-scale structural changes are not uniform, but anisotropic, with the NTSD sampling high free-energy partially structured states in equilibrium. This is observed from the varied local stabilities extracted from the emission maximum of tryptophan residues, their temperature dependence and more directly (i.e., without mutagenesis) from HDX-MS measurements. Site-specific unfolding kinetic studies highlight the rugged nature of the landscape with as many as three rate constants observed and that span nearly 600-fold depending on the location. These observations agree very well with an earlier study that reported crowded HSQC spectra for AlbAS that, remarkably, is well-dispersed in the presence of albicidin, the missing resonances for tryptophan residues W5, W27, W56, and W195 that lie either in the NTSD or at the subdomain interface (W195), and the inability to crystallize AlbAS in the apo form.^10^

The structural plasticity of AlbAS is not unique in the MerR family of transcription factors. TipAS, the thiopeptide-antibiotic sequestering protein from *Streptomyces lividans* (Figure 6A),^47^ has a conditionally disordered NTSD that binds thiostrepton.^48^ The folding–unfolding transitions associated with the NTSD in TipAS expand the ensemble and promote access to the buried site, with the CTSD acting as a scaffold (Figure 6A).^48^ On the other hand, the structural changes in AlbAS are more subtle with the partially structured states appearing as excited high free-energy states (i.e., sparsely populated) on the free-energy landscape (Figure 6B,C). The two subdomains of AlbAS resemble that of TipAS, as reported earlier;^10^ thus, a gene duplication event likely led to the evolution of a TipAS tandem repeat, which in turn accumulated mutations over the evolutionary timeline to enable the sequestration of albicidin. In fact, structural segments with low and high stability (colored and white in Figure 6D, respectively) align very well when comparing the full-length TipAS, and the two subdomains of AlbAS. Novel functions could thus evolve through a combination of gene duplication and conservation of local stability, and by extension dynamics, without regard to the actual sequence.

**Figure 6:**
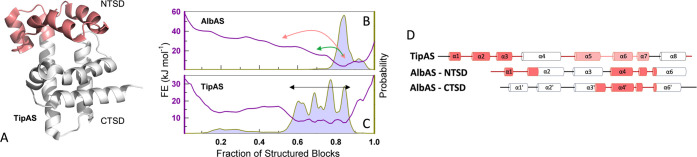
Conservation of local stability between TipAS and AlbAS. (A) The structure of TipAS (PDB id: 2MBZ) with the residues comprising the conditionally ordered N-terminal subdomain shaded in pale red. (B and C) Free energy profiles (left ordinate) against the fraction of structured blocks, and the corresponding probability distribution (right ordinate) of AlbAS (B) and TipAS (C). The arrows represent the sampling of excited states in panel (B) and the broad native ensemble in panel (C), respectively. (D) Schematic of the secondary structure alignment of TipAS and the subdomains of AlbAS. Structural segments in white and pale red indicate high and low stability regions, respectively.

Our results paint a picture in which the native ensemble of AlbAS harbors multiple substates, some of which are characterized (at a macroscopic level) by partial structure in their N-terminal subdomain. This is particularly determined by the weak thermodynamic coupling between the NTCD and CTSD that opens the structure with a time constant of ∼6 ms, as reported by kinetic studies on W195 located at the interface between the two subdomains. This large-scale intersubdomain movement potentially aids in binding albicidin by exposing the solvent-occluded binding sites. In fact, the binding of albicidin and related compounds to AlbAS stabilize it by 9–15 K while simultaneously minimizing line broadening (arising from microsecond-millisecond dynamics) in NMR experiments.^10^ Taken together with the results of the current work it is apparent that the apo form of AlbAS exhibits substantial dynamics; this in turn weakens the interactions in the native ensemble and reduces its overall thermodynamic stability, a feature which we anticipate is necessary for binding albicidin. However, it is also possible that the large dynamics of the apo form is a consequence of the loss of interdomain interactions with the coiled coil domain which sits N-terminal to the effector binding domain (Figure 1A,B). Future studies on truncated variants or comparison with the full length AlbAL both in the apo and holo forms will better answer this question.

In summary, AlbAS appears to follow a mechanism better described by conformational selection, like its cousin TipAS,^48^ illustrating how native ensemble dynamics drives function even in large protein systems associated with antibiotic sequestration. Targeting this dynamic feature could prevent the sequestration of antibiotics and would restore antibiotic efficacy, offering a promising target for drug development.

## Supplementary Material



## Notes

Thermal unfolding curves of the AlbAS variants, representative lifetime data and the mean lifetimes, urea melt of AlbAS, kinetic traces, fits and their associated residuals, comparison of deuteration extents of tryptophan harboring peptides as a function of incubation time, predictions from IUPRED and comparison with the pLDDT score from AlphaFold2, plots of apparent heat capacity as a function of protein concentration, two-state fit to the AlbAS thermogram, deuteration trends of helices and residues lining the albicidin binding tunnel (PDF)

## Abbreviations

CD: circular dichroism
bWSME: block Wako–Saitô–Muñoz–Eaton
DSC: differential scanning calorimetry
HDX-MS: hydrogen–deuterium exchange mass spectrometry
