# Supplementary material for: Local and Global Breathing Motions Prime the Access to Buried Binding Site in an Antibiotic-Sequestering Protein
Source: ACS Bio Med Chem Au. 2025 Aug 1;5(5):840–51. doi: 10.1021/acsbiomedchemau.5c00081 (PMC12531866; doi:10.1021/acsbiomedchemau.5c00081)
Supplement: Supplementary file 1 [file bg5c00081_si_001.pdf]

Supplemental Information

Local and Global Breathing Motions Prime the Access to Buried Binding Site in an Antibiotic-Sequestering Protein

Lawanya Natarajan[1], *Dmitry Loginov*[2], *Alan Kadek*[2], *Petr Man*[*,2] & *Athi N. Naganathan*[*,1]

[1]Department of Biotechnology, Bhupat & Jyoti Mehta School of Biosciences, Indian Institute of Technology Madras, Chennai 600036, India

[2]Institute of Microbiology - BioCeV, Academy of Sciences of the Czech Republic, Vestec, 252 50, Czech Republic

AUTHOR INFORMATION

Corresponding Author

e-mail: athi@iitm.ac.in, pman@biomed.cas.cz

Phone: +91-44-2257 4140

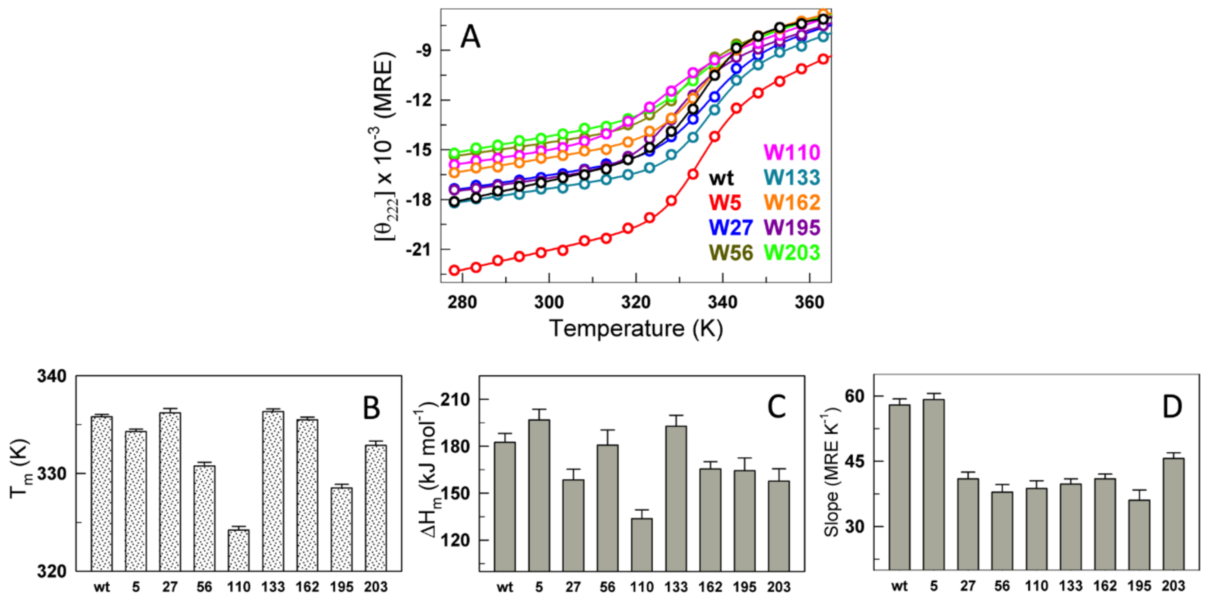

Figure S1 Single tryptophan mutants of AlbAS. (A) MRE at 222 nm from far-UV CD thermal melts of single tryptophan mutants (circles). The curves represent the fit to a two-state model. (B) Melting temperatures from a two-state fit to the melting curves. The numbers on abscissa indicate the location of the sole tryptophan in mutants. (C) Enthalpy of unfolding at the melting temperature. (D) Slopes of native baselines obtained from two-state fits to the melting curves in panel A.

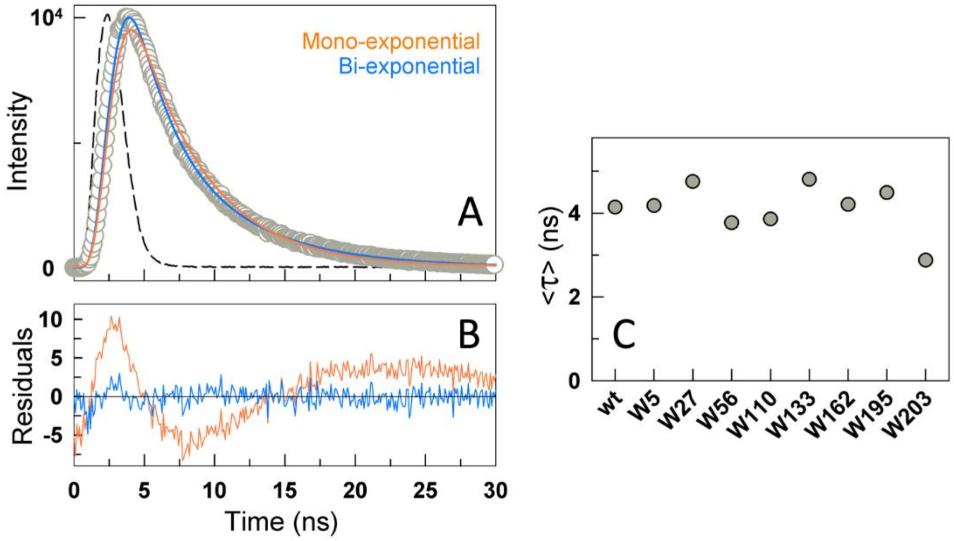

Figure S2 Tryptophan life-time measurements. (A) Intensity curve of the wt fit to mono- and bi-exponential functions. Instrument response function is shown as a dashed curve. (B) Residuals obtained from each of the fits in panel A. (C) Amplitude-weighted lifetimes of wt and mutants at 298 K.

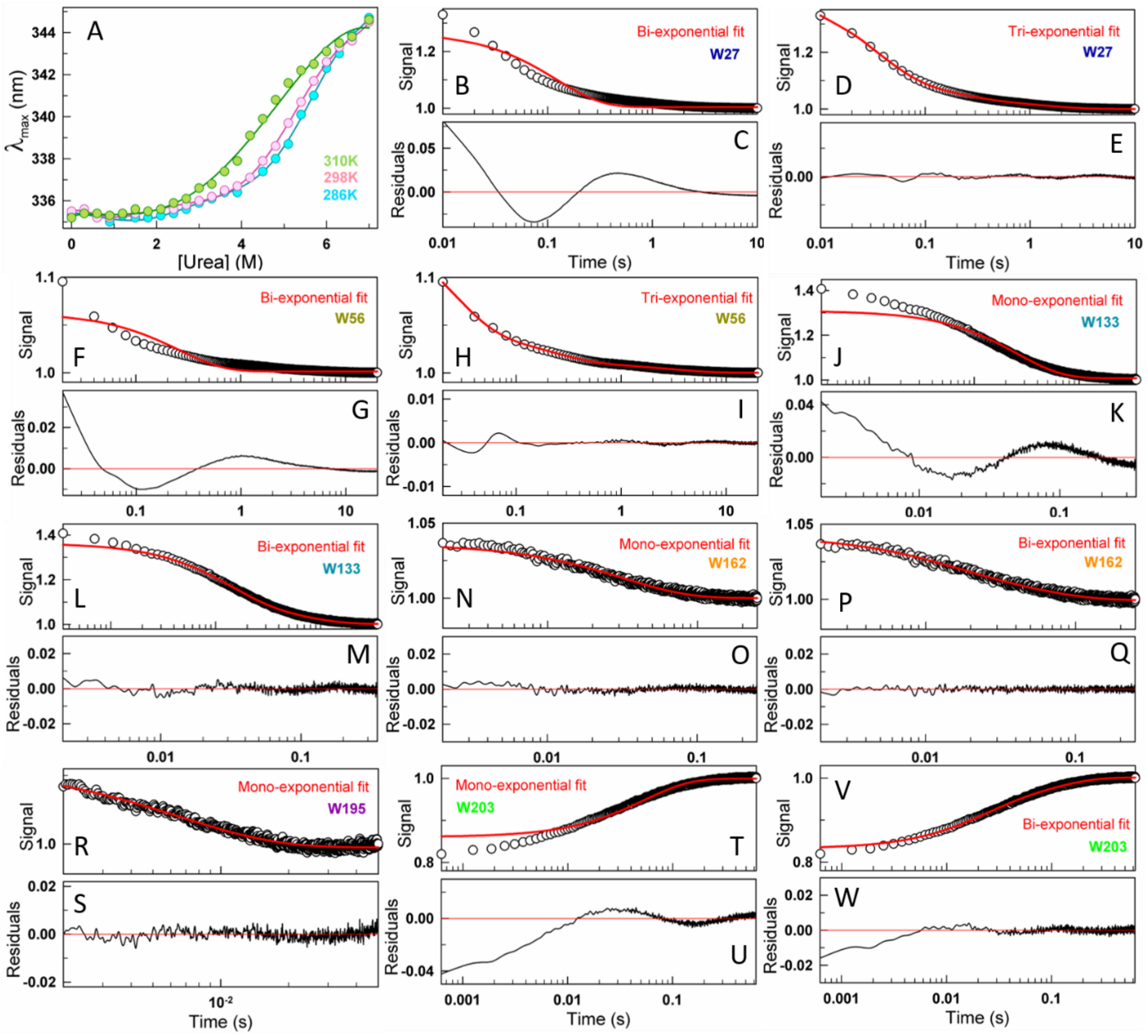

Figure S3 (A) Fluorescence emission maxima of the wt at 286, 298 and 310 K at increasing concentrations of urea (equilibrium experiment). (B – X) Relaxation traces from fluorescence stopped-flow kinetics (circles) and fits (red curves), with the residuals shown below for each of the six mutants. Signals from mutants W27 and W56 require a tri-exponential fit (B - I), while those from W133, W162 and W203 fit best to a bi-exponential function (J-Q, T-W). W195 relaxation curve is fit to a mono-exponential function (R & S).

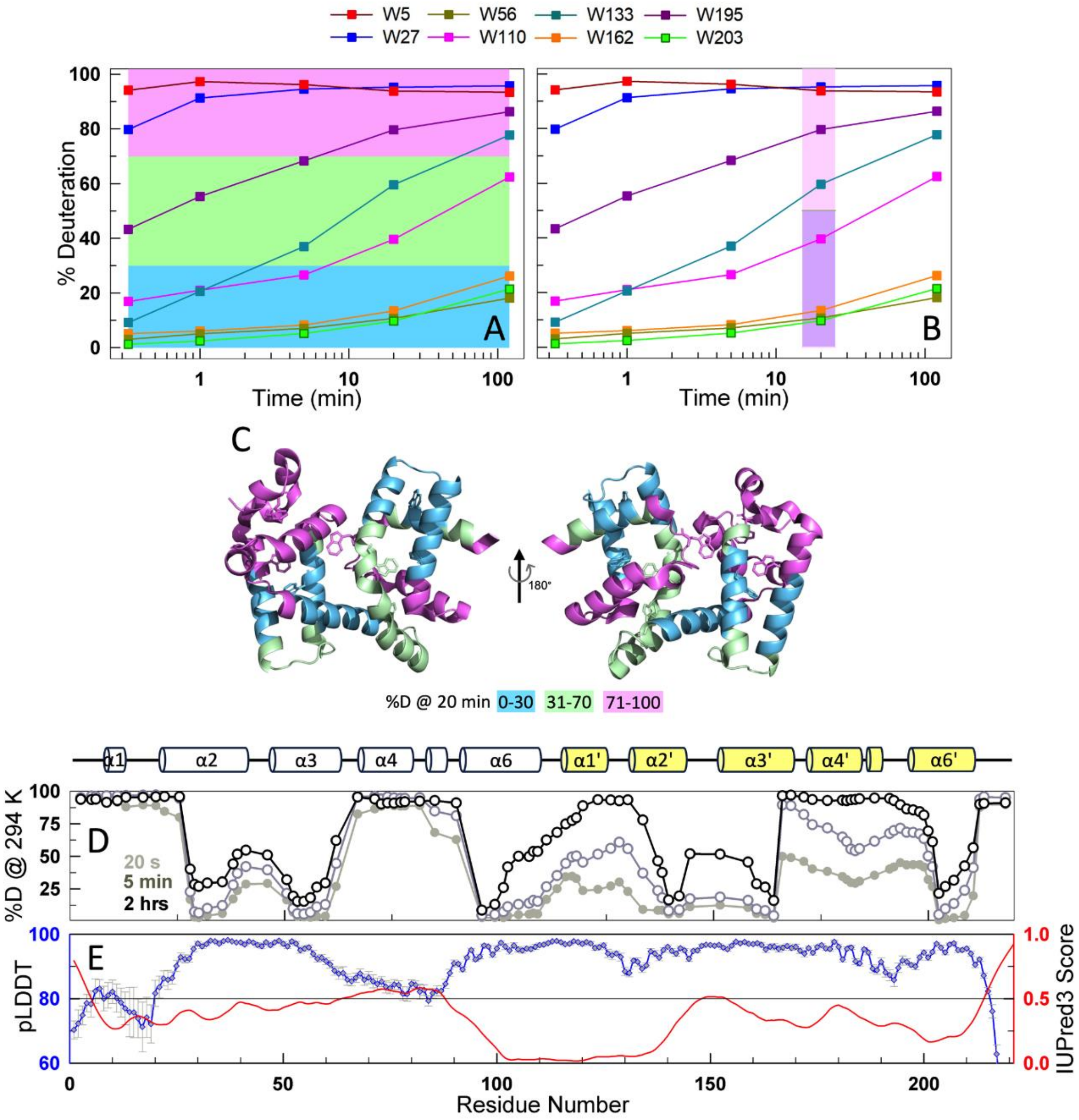

Figure S4 (A, B) Percentage deuteration (%D) of segments with tryptophan as a function of time. Panel A is demarcated into zones with low (0-30, colored blue), moderate (31-70, colored green), and high (71-100, colored pink) %D. W56, W162 and W203 fall in the blue zone. W110 and W133, start at the blue zone but transition to green and pink, respectively, with time. W195 transitions to the pink zone from green, while W5 and W27 remain in pink. The %D of segments at 20 min, highlighted with pink for %D > 50 and purple for %D < 50, compares with the trends observed for λ_{max} values of tryptophan mutants seen in Figure 2C (panel B). (C) Cartoon of AlbAS structure mapped with %D values at 20 min with color scheme (legend below) as panel A. The pink regions can be considered as exhibiting low stability (higher dynamics), while the blue regions the least dynamic, providing stability. The green regions display intermediate exchange at 20 min. (D) %D at 20 s, 5 min and 2 hr across residue number. (E) The pLDDT score (blue), a measure of tendency for disorder, indicates a more ordered CTSD compared to NTSD. The predicted disorder score from IUPred3 (red) indicates a higher disorder tendency for the most of the NTSD residues (specifically for 64-87 that fall in α4 and α5). Residues 100-140 (α1', parts of α6 and α2') are predicted to be the most ordered (low IUPRED3 score).

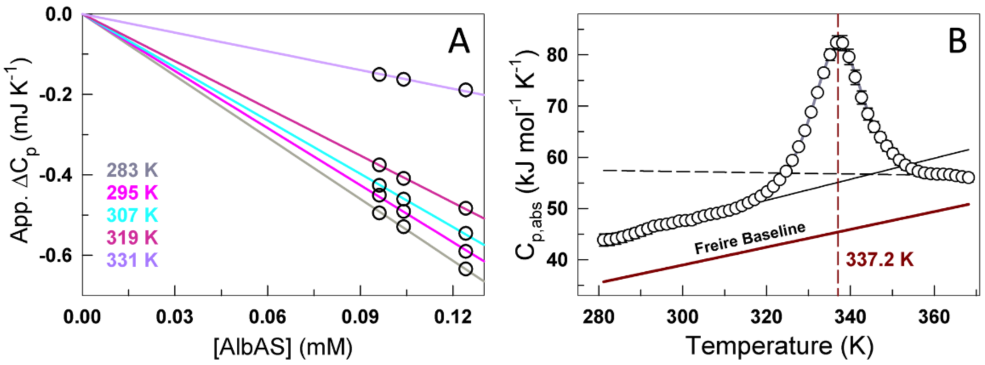

Figure S5 AlbAS is not a two-state folder. (A) Apparent heat capacity as a function of AlbAS concentration at the indicated temperatures. (B) Absolute heat capacity values (circles) derived from the apparent heat capacities fit to a two-state model (curve) along with folded (black continuous) and unfolded (black dashed) baselines. The crossing of baselines highlights that the unfolding equilibrium cannot be described by a two-state model. The vertical red dashed line indicates the melting temperature. Freire baseline represents the expected enthalpic fluctuations of a fully folded protein of the same molecular weight.

Table S1 Averaged helical deuteration profiles across peptide fragments constituting the helices. The dark horizontal line demarcates the domain boundary between NTSD and CTSD.

Helix limits	Segment start	Segment end	No. of segments	Helix name	20 s		5 min		2 hr	
					<%D>	Std.dev.	<%D>	Std.dev.	<%D>	Std.dev.
8-12	7	14	3	α1	91.15	2.54	96.48	0.13	93.10	1.61
21-41	21	42	10	α2	26.71	28.97	35.58	31.98	49.68	24.83
47-63	50	63	8	α3	8.52	8.08	14.78	12.19	27.25	14.45
68-80	71	78	6	α4	88.39	1.10	94.81	0.69	91.50	1.22
84-88	85	85	1	α5	68.15	0.00	84.66	0.00	92.72	0.00
92-110	95	109	8	α6	4.40	1.28	10.86	4.21	37.05	17.36
116-126	115	126	6	α1'	29.49	4.54	49.52	3.85	84.47	7.57
132-145	131	145	6	α2'	8.18	1.87	18.87	9.75	41.56	20.72
153-170	146	169	7	α3'	19.34	19.27	33.56	35.40	51.63	30.55
174-186	174	184	6	α4'	32.41	3.13	60.53	6.20	93.24	0.49
188-191	191	192	2	α5'	40.92	0.81	68.55	1.06	93.53	0.83
198-213	198	213	11	α6'	31.98	30.60	45.94	30.25	59.59	24.61

Table S2 Trends in deuterium exchange with solvent for residues lining the binding tunnel. Rows 1-13 and 14-26 correspond to the NTSD and CTSD, respectively. The last column (index) is colored based on %D values at 20 min (see Figure 4F in the main text).

Binding site residues	Peptide segment start	Peptide segment end	20 s	1 min	5 min	20 min	2 hr	Location	#
Y2	1	4	92.67	95.77	94.86	93.36	93.65	Loop	1
F16	15	19	89.41	96.29	96.90	95.30	95.18		2
N24	24	27	79.81	91.35	94.53	95.26	95.75	α2	3
W27	24	27	79.81	91.35	94.53	95.26	95.75		4
W56	56	56	3.05	5.04	7.02	10.73	18.15	α3	5
L60	59	60	4.01	8.06	12.88	18.83	29.48		6
L71	71	71	86.31	95.75	95.89	92.88	93.94	α4	7
L74	74	74	90.06	95.52	95.54	90.65	91.00		8
N75	75	76	88.73	94.24	94.73	90.40	90.77		9
H78	78	78	88.48	93.66	94.46	92.02	91.84		10
T88	86	94	62.74	70.93	81.05	87.82	90.98	α5	11
I95	95	97	2.89	2.50	4.68	5.82	8.62	α6	12
T99	98	100	2.94	3.00	5.32	7.80	13.07		13
L130	130	130	23.01	36.80	55.76	75.67	93.30	α2'	14
W133	131	136	9.15	20.59	37.04	59.62	77.79		15
P134	131	136	9.15	20.59	37.04	59.62	77.79		16
V137	137	137	8.47	15.71	23.65	33.87	46.35		17
W162	162	162	5.13	6.08	8.26	13.43	26.22	α3'	18
Y169	167	169	48.91	72.99	88.85	93.23	96.92		19
R181	181	181	32.07	46.36	61.33	77.46	92.68	α4'	20
G193	193	194	45.02	58.62	71.46	82.58	89.46	Loop	21
T194	193	194	45.02	58.62	71.46	82.58	89.46		22
W195	195	195	43.33	55.34	68.36	79.68	86.33		23
M196	196	197	43.51	55.23	68.27	79.46	85.50		24
L201	201	201	26.71	33.06	41.90	51.06	61.13	α6'	25
Q205	205	207	2.72	6.66	13.68	20.20	31.55		26
